# Exercise Dependence and Cardiovascular Reactivity to Acute Psychological Stress: An Extension and Replication Study

**DOI:** 10.1111/psyp.70389

**Published:** 2026-08-25

**Authors:** Anna C. Whittaker, Yuan Tian, Jenni Connelly, Jennifer L. J. Heaney, Aiden J. Chauntry, Siobhán Howard

**Affiliations:** ^1^ Faculty of Health, Sport and Society University of Stirling Stirling Scotland UK; ^2^ Clinical Immunology Services School of Inflammation, Infection and Immunology, College of Medicine and Health, University of Birmingham Birmingham England UK; ^3^ NIRSense Inc Morrisville North Carolina USA; ^4^ Department of Exercise and Sport Science University of North Carolina at Chapel Hill Chapel Hill North Carolina USA; ^5^ Department of Psychology, SASH Lab, Centre for Social Issues Research, Faculty of Education and Health Sciences University of Limerick Limerick Ireland; ^6^ Health Research Institute University of Limerick Limerick Ireland

**Keywords:** acute stress, cardiovascular reactivity, exercise dependence

## Abstract

Low cardiovascular reactions to acute psychological stress have been shown among those with substance and non‐substance dependencies like exercise dependence. However, associations between exercise dependence and low cardiovascular reactions to stress have been demonstrated between small sex‐specific groups with extreme exercise dependence scores. The present study sought to replicate previous findings as well as examine whether exercise dependence across the range of scores was linearly related to reactivity magnitude in a more diverse sample. Participants (*n* = 161, 65% female) undertook laboratory stress testing. Cardiovascular activity was measured at rest and in response to a 10‐min Paced Auditory Serial Addition Test (PASAT). Exercise dependence was measured via three validated questionnaires. There were no significant linear associations between exercise dependence scores and blood pressure reactivity, with or without adjustment for covariates. In a subsample of females aged < 30 years, small negative associations emerged for blood pressure reactivity. Replication analyses of extreme groups created using the EABQ, as in prior work, showed no significant differences in reactivity, but there were some indications of low reactivity among individuals classified as exercise dependent on the EDQ. In conclusion, differences in stress reactivity may not be confined to extreme scoring groups and can also emerge as linear associations. However, the presence and form of these effects appear to be measure‐, outcome‐ and sample‐dependent.

## Impact Statement

This study extends research on exercise dependence and low cardiovascular and cortisol reactivity to acute psychological stress. It shows, for the first time, that exercise dependence links with low stress reactivity are not confined to extreme comparison groups but are also evident linearly. Thus, blunted stress reactivity is apparent at lower levels of dependence than previously thought. The implications for future research and practice are to explore other linear associations with reactivity as well as comparing extreme groups to identify those at risk of a range of negative correlates of low reactivity. If the peripheral cardiovascular changes associated with addictive or dependent behavior can occur at lower levels than previously considered, then this suggests that dysregulation at the brain level is also occurring at these lower levels of dependence. This may increase the risk of other unhealthy behaviors or outcomes. This could form the basis for interventions attempting to reverse these changes, using cardiovascular stress reactivity as a noninvasive peripheral marker.

The cardiovascular reactivity hypothesis (Obrist 1981) spawned several decades of research into exaggerated stress responses to acute psychological stress and cardiovascular disease (see e.g., Lovallo and Gerin 2003). Consequently, for many years, it was considered that low or blunted reactivity to acute stress was protective to health. More recent research has challenged this notion, with accumulating evidence now suggesting that low or blunted reactivity to stress is also linked to negative, albeit mainly non‐cardiovascular, health outcomes (for reviews see Carroll et al. 2009, 2017; Lovallo 2011; Phillips et al. 2013; Whittaker et al. 2021). The largest samples demonstrating these associations have included the West of Scotland 20‐07 study and Dutch Famine Birth Cohort Study (which also included and replicated effects for cortisol reactivity). Both of these samples also show the typical positive associations between exaggerated reactivity and future cardiovascular disease (e.g., Carroll et al. 2011, 2012). Over the past 20 years, in these studies and others, a range of links between low reactivity and negative health and behavioral outcomes have been observed, such as depression and anxiety (Carroll et al. 2007; de Rooij et al. 2010; Phillips et al. 2011), self‐rated health (de Rooij and Roseboom 2010; Phillips, Der, and Carroll 2009) and cognitive ability (Ginty et al. 2011a, 2011b) among other outcomes and clusters of health and psychosocial conditions/exposures (e.g., Keogh et al. 2023).

It has been proposed that these health and behavioral correlates of blunted or low reactivity indicate altered functioning of frontolimbic brain areas essential for motivation and behavioral regulation, which has been termed “motivational dysregulation” (Carroll et al. 2009; Ginty et al. 2014; Lovallo 2011). Interestingly, these brain areas are also involved in autonomic nervous system regulation (Lovallo 2005; Lovallo et al. 2013) and can contribute to impulsive behaviors and impaired response inhibition (Allen et al. 2009; Bennett et al. 2014; Bibbey et al. 2016; Muñoz and Anastassiou‐Hadjicharalambous 2011). Consequently, it is not surprising that such dysregulation has also been found to relate to behavioral characteristics which require psychological effort, such as perseverance in unsolvable puzzle tasks (Chauntry et al. 2019; Whittaker and Chauntry 2021), study participation (Ginty et al. 2015), giving up smoking (Al'Absi et al. 2005) and social participation (John‐Henderson et al. 2019).

Blunted reactivity has also been associated with a range of addiction‐related behaviors, such as smoking (Al'Absi 2018; Al'Absi et al. 2003; Ginty et al. 2014, 2016; Girdler et al. 1997; Kirschbaum et al. 1993, 1994; Rohleder and Kirschbaum 2006; Roy et al. 1994; Sheffield et al. 1997; Straneva et al. 2000), alcohol (Jones et al. 2013; Lovallo 2006; Panknin et al. 2002; Sorocco et al. 2006) and obesity (Carroll et al. 2008; Phillips et al. 2012). Some early critics of the research on blunted reactivity and addiction behaviors cited the potential impact of lower effort or engagement, such as with forced abstinence, with tasks to explain possible blunting effects. However, it has also been shown that individuals with low or blunted responses to psychological stressors do not show lower appraisals of task stressfulness or difficulty, objective task effort, or a physiological capacity to respond to other non‐psychological laboratory challenges compared to exaggerated responders (Brindle et al. 2017). Others claimed that substances, such as tobacco, might directly blunt reactivity to explain these effects. However, this is deemed unlikely as low cardiovascular reactivity has been observed in smokers both with and without a nicotine replacement patch (Girdler et al. 1997).

It has also been demonstrated that low or blunted reactivity, including cardiovascular and cortisol reactivity, seems to be a characteristic also associated with non‐substance addictions, such as disordered eating, for example, symptoms of bulimia (Ginty et al. 2012) and exercise dependence (Heaney et al. 2011). However, these studies often demonstrated the link between addictive or dependent behaviors and reactivity through comparing small groups of those at the extreme of dependence and non‐dependence, rather than across the range of scores for both dependency and reactivity, making their conclusions preliminary. Heaney et al. (2011) demonstrated lower heart rate (HR) and cortisol reactivity among 10 women (as exercise dependence is most common in women [Bamber et al. 2000]) with extremely high exercise dependence scores compared to 10 women with scores of zero. These associations withstood adjustment for covariates such as physical fitness, comorbid eating disorders and stress task performance, which might be expected to interact with either exercise dependence or reactivity. Exercise dependence can be operationalized as beliefs about exercise in the context of excessive exercise (measured by the Exercise Beliefs Questionnaire (EBQ)) s (Loumidis and Wells 1998), behavioral features consistent with dependence/addiction (using the Exercise Dependence Questionnaire [Ogden et al. 1997]), or attitudes/behaviors toward exercise involvement (though the Exercise Attitudes and Behaviors Questionnaire [EABQ] [Bamber et al. 2003]). These instruments may capture overlapping but non‐identical constructs, which could contribute to variability in observed associations with physiological stress responding. However, given the design of these studies, replication is essential before drawing firm conclusions.

Consequently, the present research sought to extend and replicate the previous reactivity and exercise dependence study (Heaney et al. 2011) in a larger mixed‐sex sample with exercise dependence scores across the continuum, rather than a focus on the two groups at the extremes. The aim was to examine whether cardiovascular reactivity related to exercise dependence across the range of scores (rather than through comparison of extreme groups) and in a more diverse sample. A second aim was to extend and replicate the work of Heaney et al. (2011) by examining just younger females in continuous analyses as well as a comparison of extreme groups. It was hypothesized that cardiovascular reactivity would relate negatively to exercise dependence and this would be shown linearly as well as between extreme groups.

## 1 Method

### 1.1 Participants and Design

This study recruited 161 students from the University of Stirling, Scotland. A cross‐sectional design was used to explore the association between exercise dependence across a range of scores and physiological reactivity to acute psychological stress. Dependent variables were systolic and diastolic blood pressure, HR and cortisol reactivity to acute stress. The study was approved by the Non‐Invasive, Clinical and NHS Research Ethics Committee of the University of Stirling (NICR 19_20 [081]). All participants provided written informed consent. Data and code are available from the authors and will be archived in the University of Stirling STORRE with the manuscript following publication.

### 1.2 Measures

#### 1.2.1 Questionnaires

##### 1.2.1.1 Exercise Dependence

The EBQ (Loumidis and Wells 1998) was used to assess exercise dependency beliefs. It consisted of 21 items measuring four factors of social desirability, physical appearance, mental and emotional functioning andvulnerability to disease and aging. The questionnaire described thoughts of exercise, with answers to each item being from 100 indicating complete agreement to 0 indicating complete disagreement. Items such as “If I do not exercise my brain will become unhealthy.” Higher scores indicated greater support for the belief that not exercising has negative consequences. In the questionnaire's original validation, the Cronbach's alpha coefficients were all greater than 0.70 andit had good retest reliability (Loumidis and Wells 1998). Reliability in the present study was Cronbach's alpha = 0.93.

The Exercise Dependence Questionnaire (EDQ) (Ogden et al. 1997) was used to assess addictive exercise behaviors. It consisted of 29 items measuring eight factors of interference with social/family/work life, positive reward, withdrawal symptoms, exercise for weight control, insight into problems, exercise for social reasons, exercise for health reasons andstereotyped behaviors. It used a 7‐point scale from 1 (strongly disagree) to 7 (strongly agree) to indicate the level of agreement on each statement. Items included questions such as “my pattern of exercise interferes with my social life.” Higher scores indicated higher levels of exercise dependence behavior. The original validation study showed the overall Cronbach's alpha coefficient was 0.84 and the scale had good retest reliability (Ogden et al. 1997). Internal consistency in the present study was Cronbach's alpha = 0.88.

The Exercise Attitudes and Behavior Questionnaire (EABQ) (Bamber et al. 2003) was used to assess people's feelings and behaviors about exercise. It consisted of 13 items and used a 7‐point scale from 0 (definitely no) to 6 (definitely yes) to indicate the level of agreement on each statement. Items included prompts such as “I am constantly thinking about exercise.” Higher scores indicated stronger attitudes towards exercise and greater dependence on exercise. Cronbach's alpha coefficient was previously 0.88 (Heaney et al. 2011) and in the present study was 0.84.

##### 1.2.1.2 Disordered Eating

The SCOFF (sick‐control‐one‐fat‐food) scale (Morgan et al. 1999) was used to assess disordered eating as this is often comorbid with exercise dependence behaviors. It consisted of five items focusing on anorexia and bulimia. Items were scored with 0 (no) and 1 (yes), where higher scores indicated higher risk of eating disorders. Items such as “Do you make yourself Sick because you feel uncomfortably full?” The sensitivity of the questionnaire was 0.80 and the specificity was 0.93 (Morgan et al. 1999). Reliability in the present study was 0.68, as the items are distinct behaviors.

##### 1.2.1.3 Stress Task Ratings

The stress task self‐report rating scales were used to measure how participants felt about the stress test and included an indicator of task engagement as used in previous studies (e.g., Heaney et al. 2011). These consisted of seven items on a 7‐point scale from 0 – not at all to 6 – extremely. Questions assessed perceived difficulty, stress, arousal, performance, confusion, engagement and embarrassment.

##### 1.2.1.4 Socio‐Demographics and Health Behaviors

The socio‐demographic and anthropometric information collected included height, weight, sex, age, ethnicity, postal code, wake‐up time on the experiment day, any previous experience of stress test participation, existence of chronic disease, continuous medication use except the contraceptive pill and highest parental occupation as an indicator of socio‐economic status. Parental occupations were classified using the Standard Occupational Classifications (SOC 2000—Office for National Statistics) across seven occupational groups: professional, managerial/technical, skilled non‐manual, skilled manual, partly‐skilled, unskilled and armed forces and then recoded into manual and non‐manual occupations. If participants were female, questions also included oral contraceptive pill use (yes/no) and date of last period to indicate menstrual cycle phase.

Health behaviors were measured via questions used in the Whitehall II study (Marmot and Brunner 2005). These included questions about smoking and alcohol intake frequency and amount (units) and sleep duration. Estimated physical fitness was measured by a standardized questionnaire (Jurca et al. 2005) assessing physical activity levels divided into one of five levels (higher level equals more intense activity). This was then used to calculate metabolic equivalents (METS) in combination with participants' age, sex and resting HR (Jurca et al. 2005).

#### 1.2.2 Psychological Stress Test

The study used a modified 10‐min version of the Paced Auditory Serial Addition Task (PASAT) (Gronwall 1977), with additional social evaluation and competition as established previously in many studies (e.g., Brindle et al. 2017; Whittaker and Chauntry 2021). A series of single‐digit numbers, from one to nine, were randomly presented via a CD. Participants were required to memorize the previous number they heard and add it to the new number they heard and verbally state the answer. The presentation rate of the numbers increased as the test went on; a total of 200 numbers were presented and the interval between each digit was 4.5 s for the first 2 min and then 0.5 s shorter every 2 min afterward. The experimenter stood 1 m in front of the participant and obtrusively marked their responses as correct or missing/incorrect to increase social evaluation. Prior to formally starting the PASAT, participants were given the opportunity for a brief practice. After the practice, to increase feelings of competition, participants were instructed that (1) they were in competition with other students as shown on a leaderboard in front of them, (2) that the numbers would speed up as the test went on and that they must keep up with the numbers. To increase feelings of social evaluation, they were also instructed that they needed to look at the large TV screen directly in front of them that showed their live performance and that this would be video recorded for body language and anxiety assessment by “experts.” They were specifically asked to do their best and pick up the pattern again as soon as possible if they lost it and were told if concentration or performance was bad, scores would drop rapidly. They were also told that the experimenter would sound a loud buzzer every time they gave an incorrect answer, looked away from the screen, stuttered, mumbled, or hesitated. In fact, they were not video‐recorded and the buzzer was sounded a standardized number of times in line with an error or hesitation wherever possible—this was included in Debriefing information post‐testing for ethical reasons. The test was also scored out of a total of 1000 points, with 5 points deducted for each missed or incorrect answer.

#### 1.2.3 Cardiovascular Measures

Systolic blood pressure (SBP), diastolic blood pressure (DBP) and HR were measured discontinuously by semi‐automatic sphygmomanometer (Critikon, Hoofddorp). This was placed on the upper arm of the participant's dominant hand.

#### 1.2.4 Salivary Cortisol

Saliva was collected via Salivettes (Sarstedt, Germany) by asking participants to place a cotton swab in their mouths and chew it for 1 min before replacing it in the Salivette tube. Following testing, the Salivettes were centrifuged (Thermo, Waltham) at 4000 rpm for 5 min at room temperature. The separated saliva was then pipetted into two separate microtubes and stored in two freezers at −20°C. Salivary cortisol samples were analyzed in duplicate by ELISA (Salimetrics, Stratech Scientific Limited., UK) using the competition principle and microplate separation. Samples containing an unknown amount of cortisol and a fixed amount of cortisol conjugated with horseradish peroxidase competed for the binding sites of antibodies coated onto a 96‐well plate. The microplate was washed an hour later to stop the competition reaction. After adding the substrate solution, the cortisol concentration was inversely proportional to the optical density measured at 450 nm. The mean intra‐assay and inter‐assay coefficients of variation were < 10%.

### 1.3 Procedure

Participants were recruited through verbal and poster advertising in classes and clubs at the University of Stirling. Participants booked a time slot after 1 pm by scanning a QR code on the poster or sending an email and leaving their name and email address so that the experimenter could send them the participant information sheet. The day before testing, the experimenter sent an email to participants reminding them not to consume caffeine, eat, or smoke/vape in the 2 h before the testing, or exercise vigorously for 4 h or consume alcohol in the 12 h before testing; time of testing was also recorded, with all sessions being held in the afternoon. On the afternoon of the experiment, the participant sat in a semi‐supine position in a phlebotomy chair, gave informed consent, then had the blood pressure cuffs attached. During a 10‐min adaptation period, participants completed the Dependence questionnaires above, followed by a formal 10‐min baseline period. Blood pressure and HR were measured using the sphygmomanometer at 2 and 6 min during the formal baseline, stress task and recovery period. Participants were given the first Salivette in the final minute of baseline, immediately after the stress task and in the final minute of the 20‐min recovery period. Following baseline, the experimenter put on a white lab coat and started the PASAT description. After the participant did the practice task, the experimenter continued to read out the PASAT instructions, after which the stress task was formally started. During the post‐stress recovery period, participants were asked to continue to complete the questionnaire pack, including PASAT ratings. At the end of recovery, participants were given a debriefing and thanked for their participation. The experimenter entered all the questionnaire data into SPSS and processed and stored the saliva samples on the same day.

### 1.4 Data Analysis

The statistics programme SPSS v.30 (IBM, USA) was used for data analysis. The data analysis in this study categorized ethnicity into white and non‐white and occupation status into manual and non‐manual. Body mass index (BMI) was calculated as weight/(height × height) in kg/m^2^ from direct laboratory measurement. SBP, DBP and HR during the baseline, stress and recovery periods were obtained by calculating the average values during baseline, stress and recovery. SBP, DBP and HR reactivity were calculated as the average stress values minus the average baseline values. Cortisol reactivity was calculated as sample three at the end of recovery (corresponding to the peak response) minus the baseline sample. Repeated‐measures ANOVAs were used to test whether the PASAT altered blood pressure, HR and cortisol. Pearson correlation analyses were used to examine the unadjusted associations between exercise dependence scores and SBP, DBP, HR and cortisol reactivity. For the primary analysis, adjusted models were run as a series of linear regression models by entering covariates first in the model followed by each exercise dependence score as the independent variable, with reactivity as the dependent variable. PASAT score and baseline cardiovascular/cortisol level were added as covariates, decided a priori, into all adjusted models to account for task effort and resting cardiovascular function. Additional important covariates known to commonly relate to either exercise dependence (disordered eating, exercise engagement, estimated fitness) or reactivity (age, gender, menstrual cycle phase, health behaviors, PASAT ratings) were explored through one‐way ANOVAs (categorical variables) or correlations (continuous variables). For the secondary replication and extension analyses of Heaney et al. (2011), linear analyses were repeated just in young females aged ≤ 30 years as well as extreme groups analyses comparing the 10 (or as close as the distribution of scores would allow) highest and lowest scorers on the EABQ, using ANOVA and then ANCOVA to adjust for covariates. Finally, a sensitivity analysis using the EDQ cut‐off of 116 or higher (Ogden et al. 1997) to indicate scoring above the recommended cut‐off suggestive of exercise dependence risk was also used to compare reactivity between those with and without dependence using this criterion. Variations in degrees of freedom reflect occasional missing data.

## 2 Results

### 2.1 Participants

In the sample, there was a total of 161 participants of which 105 (65.2%) were female with a mean (SD) age of 22.2 (4.9) years. In terms of ethnicity, 133 (82.6%) described themselves as “white”, 16 (9.9%) as “Asian”, 2 (1.2%) as “black” and 10 (6.2%) as “other”. Nineteen percent were from a manual occupational household based on their parental occupation. Twelve (7.5%) reported having chronic diseases, most commonly asthma and 24 (14.9%) were taking ongoing medication, mainly for asthma, anxiety, or depression.

To replicate the previous study of young females, a subsample (*n* = 102) of women aged ≤ 30 years was also examined with a mean (SD) age of 21.1 (1.99) years. In terms of ethnicity, 87 (85.2%) described themselves as “white”, 8 (7.8%) as “Asian”, 1 (0.9%) as “black” and 6 (5.9%) as “other”. Twenty‐one percent were classified as from a manual occupational household based on their parental occupation. Nine (8.8%) reported having chronic diseases, most commonly asthma and 20 (19.6%) were taking ongoing medication, mainly for asthma, anxiety, or depression. The descriptive statistics for socio‐demographics, anthropometrics, questionnaire scores and reactivity values are shown in Table 1.

**TABLE 1:** Descriptive statistics for full and subsample (mean[SD]).

Variable (units)	Full sample (*n* = 161)	Subsample (*n* = 102)
Sex (female)	105 (65.2)	102 (100%)
Age (years)	22.2 (4.9)	21.1 (1.99)
Ethnicity (white)	133 (82.6)	87 (85)
Occupational group (manual)	31 (19.3)	21 (21)
Taking contraceptive pill (yes)	36 (22.4)	35 (34)
Menstrual cycle phase (follicular)	27 (16.8)	27 (26.5)
Chronic illness (yes)	12 (7.5)	9 (9)
Taking medication (yes)	24 (14.9)	20 (20)
BMI (kg/m^2^)	24.4 (3.60)	24.0 (3.74)
Estimated Fitness (METS)	17.0 (1.74)	16.3 (1.21)
Smoker (yes)	24 (14.9)	16 (16)
Sleep (*≥* 8 h)	59 (36.6)	37 (36)
Drinks alcohol (yes)	120 (74.5)	75 (74)
SCOFF total score	0.9 (1.23)	1.1 (1.33)
EABQ score	30.5 (13.13)	28.1 (12.90)
EDQ score	104.7 (23.32)	105.6 (23.69)
EBQ score	1004.0 (391.06)	977.5 (387.64)
PASAT score	609.3 (140.98)	577.7 (140.30)
PASAT Difficulty rating	4.6 (0.94)	4.6 (0.93)
PASAT Stressfulness rating	4.4 (1.32)	4.7 (1.15)
PASAT Arousing rating	2.7 (1.64)	2.6 (1.56)
PASAT Performance rating	2.3 (1.37)	2.0 (1.39)
PASAT Confusing rating	3.6 (1.54)	3.6 (1.51)
PASAT Engaging rating	4.1 (1.35)	4.0 (1.32)
PASAT Embarrassing rating	3.2 (1.93)	3.5 (1.92)
SBP reactivity (mmHg)	13.7 (11.45)	12.2 (10.88)
DBP reactivity (mmHg)	9.7 (8.90)	9.7 (7.49)
HR reactivity (bpm)	12.9 (10.34)	14.3 (10.55)
Cortisol reactivity (ug/dL) (*n* = 56)	−0.002 (0.09)	0.003 (0.09)

One participant could not contribute reactivity data due to failure to capture baseline BP/HR (*n* = 160 in total sample and *n* = 101 in young female sample). For cortisol, due to restricted funding, data were available for *n* = 77 in the full sample and *n* = 56 among the subsample.

### 2.2 Manipulation Check

**FIGURE 1:**
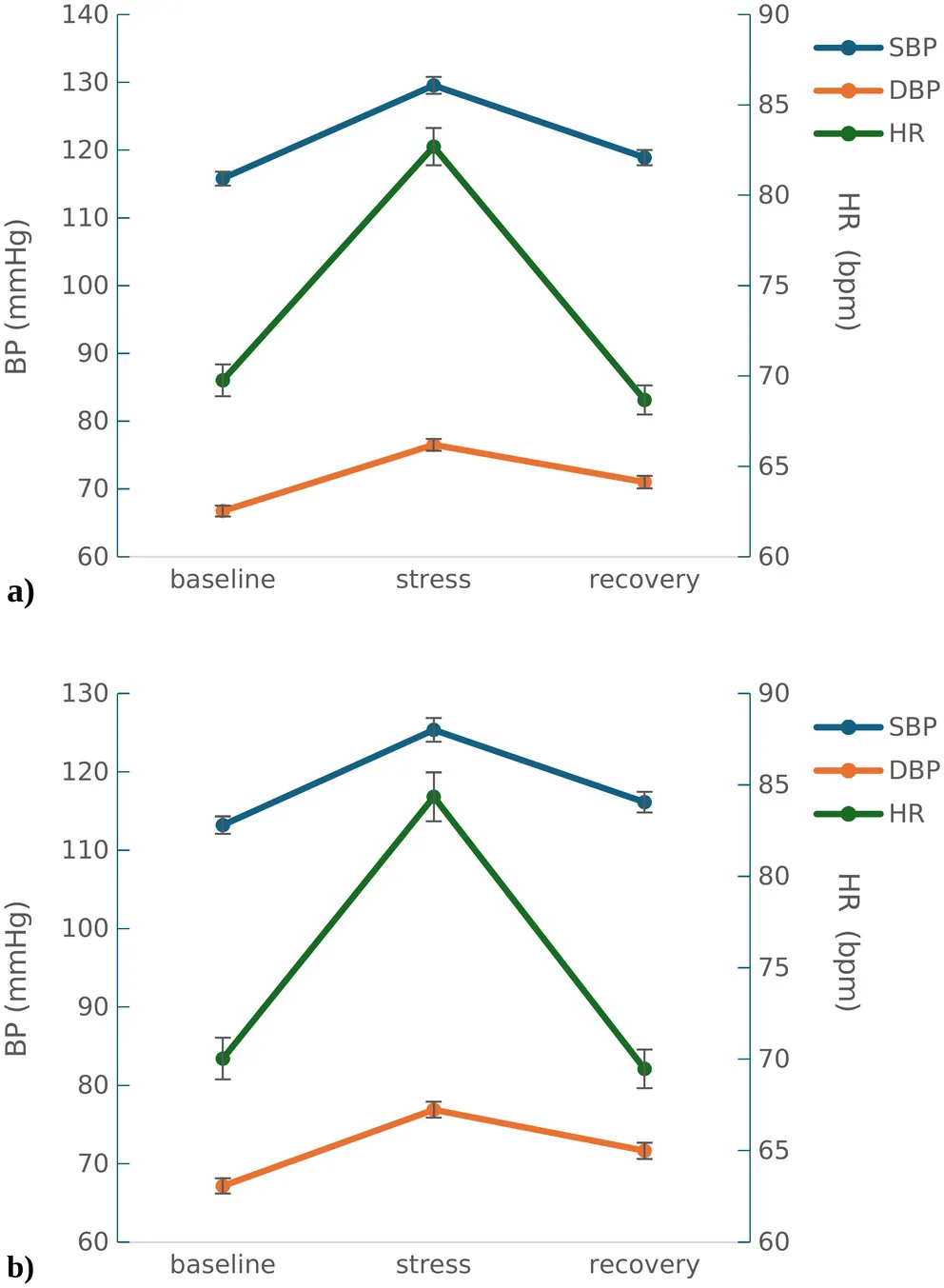
Mean (SE) cardiovascular levels across the session, (a) full sample *n* = 160, (b) young female subsample only *n* = 101.

**FIGURE 2:**
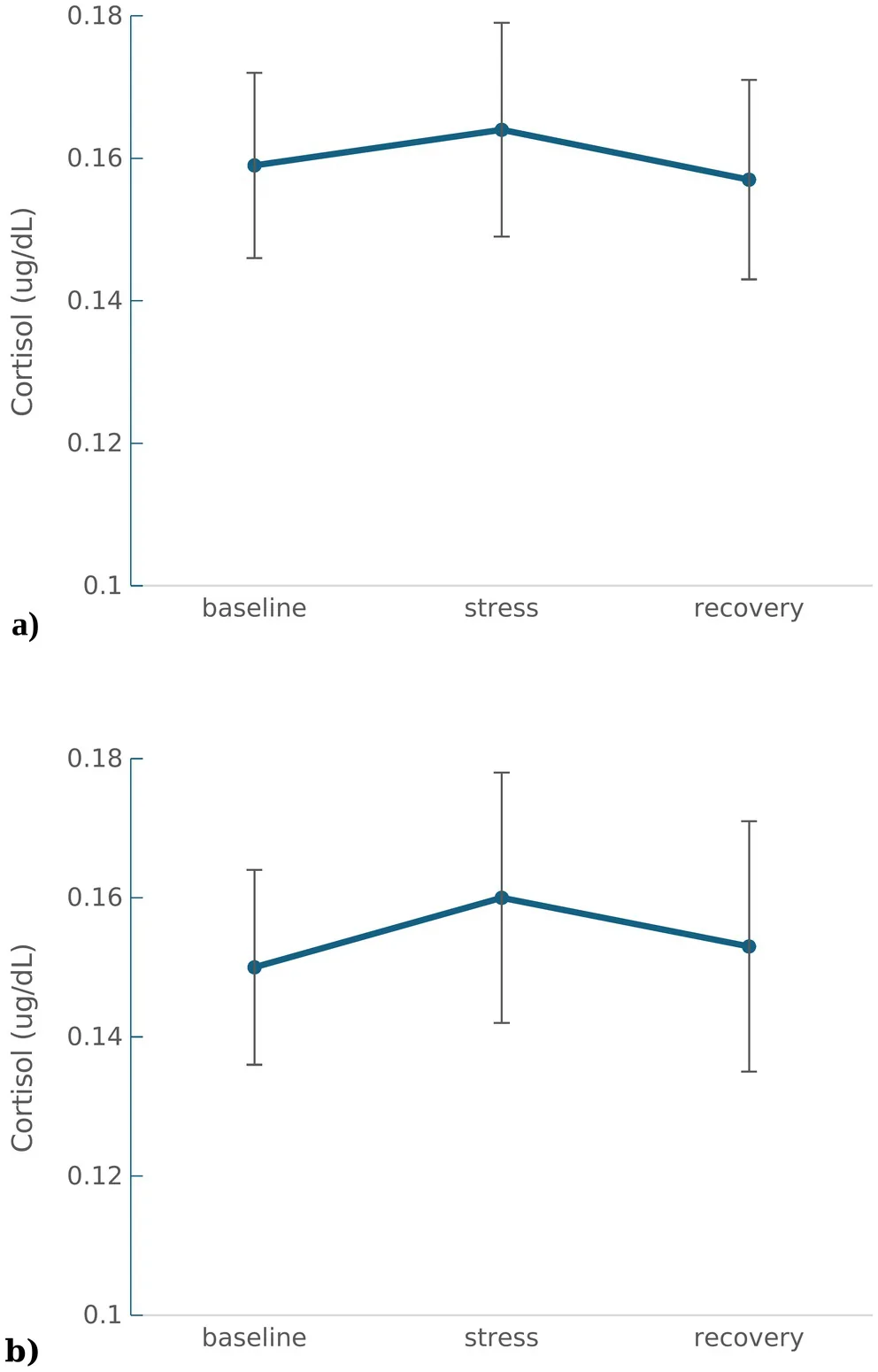
Mean (SE) cortisol levels across the session, (a) full sample *n* = 77, (b) young female subsample only *n* = 56.

To check that the PASAT was effective at perturbing physiological activity, repeated measure ANOVAs were run for SBP, DBP, HR and cortisol. There were main effects of time for SBP, *F*(2,158) = 124.80, *p* < 0.001, η^2^ = 0.612, DBP, *F*(2,158) = 103.13, *p* < 0.001, η^2^ = 0.566 and HR, *F*(2,158) = 192.91, *p* < 0.001, η^2^ = 0.709, such that each cardiovascular measure increased from baseline during stress phase and then returned towards baseline during recovery. These effects are shown in Figure 1a. For cortisol, there was no significant effect of time, *F*(2,75) = 0.582, *p =* 0.56, η^2^ = 0.015 (see Figure 2a).

In the young female subsample, there were also main effects of time for SBP, *F*(2,99) = 67.51, *p* < 0.001, η^2^ = 0.577, DBP, *F*(2,99) = 85.70, *p* < 0.001, η^2^ = 0.634 and HR, *F*(2,99) = 136.38, *p* < 0.001, η^2^ = 0.734, such that each cardiovascular measure increased from baseline to during stress and then returned towards baseline during recovery. These effects are shown in Figure 1b. For cortisol, there was again no significant effect of time, *F*(2,54) = 0.98, *p =* 0.38, η^2^ = 0.035 (see Figure 2b).

Consequently, associations with cortisol reactivity were not further explored as the task did not elicit significant stress reactivity in the sub‐groups where this was measured.

### 2.3 Unadjusted Analyses: Exercise Dependence and Reactivity

To examine associations between exercise dependence scores and reactivity, correlations were run for each scale and each reactivity score. As shown in Table 2, there were no significant relationships in the full sample.

**TABLE 2:** Unadjusted correlations (*r*) or adjusted regressions (*β*) between exercise dependence and reactivity.

	SBP reactivity	DBP reactivity	HR reactivity
**Unadjusted**			
Full sample			
EABQ score	0.05	−0.11	0.03
EDQ score	−0.12	−0.13	0.04
EBQ score	−0.03	−0.09	−0.01
Young females			
EABQ score	−0.10	−0.09	0.08
EDQ score	−0.19^#^	−0.16	0.03
EBQ score	−0.23*	−0.23*	−0.05
**Adjusted**			
Full sample			
EABQ score	0.11	−0.08	0.02
EDQ score	−0.11	−0.14	−0.05
EBQ score	−0.004	−0.08	−0.09
Young females			
EABQ score	−0.08	−0.09	0.03
EDQ score	−0.16	−0.16	−0.08
EBQ score	−0.27*	−0.22*	−0.12

* *p* = < 0.05, ^#^indicates non‐significant trend *p* = 0.06.

In the young female subsample, there were significant linear associations between EBQ scores and reactivity, such that those with higher scores had lower SBP, *r*(99) = −0.23, *p* = 0.02 and DBP, *r*(99) = −0.23, *p* = 0.02, reactivity; and for the EDQ, higher scorers showed a trend for lower SBP reactivity, *r*(99) = −0.19, *p* = 0.06 (Table 2).

### 2.4 Covariates Relating to Reactivity

In the full sample, PASAT score significantly related to SBP reactivity (positively correlated, *p* = 0.01), but was entered into all adjusted models to adjust for task effort. DBP and HR reactivity related significantly negatively to their own baseline values (*p* < 0.001 and 0.002, respectively) which provides justification for including baseline cardiovascular values in all adjusted models. Additional covariates identified as significantly relating to reactivity were: for SBP, smoking (lower in smokers, *p* = 0.03), age (positively correlated, *p* = 0.007) and PASAT performance rating (positively correlated, *p* = 0.008); for DBP reactivity, estimated fitness (negatively correlated, *p* = 0.02) and PASAT arousing rating (positively correlated, *p* = 0.03); and for HR reactivity: gender (higher in females, *p* = 0.02), drinking alcohol (lower in those who drink, *p* = 0.03), estimated fitness (negatively correlated, *p* = 0.04) and PASAT stressfulness rating (positively correlated, *p* = 0.03).

In the subsample, PASAT score only significantly positively related to SBP reactivity (*p* = 0.02) but was entered into all adjusted models to adjust for task effort. DBP and HR reactivity were significantly negatively related to their own baseline values (*p* = 0.002 and 0.01, respectively) and again were entered into all adjusted models. A further covariate identified as significantly relating to HR reactivity was drinking alcohol (lower in those who drink, *p* = 0.02). SBP and DBP reactivity did not relate to any of the socio‐demographic or health behavior variables. Correlation *r*‐values and ANOVA F values are shown in Table S1.

### 2.5 Covariates Relating to Exercise Dependence

In the full sample, covariates identified as significantly relating to exercise dependence scores were: for the EABQ, gender (higher in males, *p* = 0.003); smoking (higher in non‐smokers, *p* = 0.04), SCOFF total (positively correlated, *p* = 0.04), estimated fitness (positively correlated, *p* < 0.001), PASAT score (positively correlated, *p* = 0.002), PASAT arousing rating (positively correlated, *p* = 0.009) and PASAT confusing rating (negatively correlated, *p* = 0.003). For the EBQ, associated variables were SCOFF total (positively correlated, *p* = 0.006), estimated fitness (positively correlated, *p* = 0.02), PASAT arousing rating (positively correlated, *p* < 0.001) and PASAT confusing rating (negatively correlated, *p* < 0.001). For the EDQ, related variables were ethnicity (higher for white, *p* = 0.04), smoking (higher in non‐smokers, *p* = 0.02), age (negatively correlated, *p* = 0.005), SCOFF total (positively correlated, *p* = 0.03), estimated fitness (positively correlated, *p* < 0.001), PASAT stressfulness rating (positively correlated, *p* = 0.03), PASAT arousing rating (positively correlated, *p* = 0.003), and PASAT confusing rating (negatively correlated, *p* = 0.01).

In the subsample, covariates identified as significantly relating to exercise dependence scores for the EABQ were, estimated fitness (positively correlated, *p* < 0.001). For the EBQ, associated variables were SCOFF score (positively correlated, *p* = 0.02), ethnicity (higher for white, *p* = 0.01), PASAT arousing rating (positively correlated, *p* = 0.004) and PASAT confusing rating (negatively correlated, *p* = 0.006). For the EDQ, related variables were age (negatively correlated, *p* = 0.04), ethnicity (higher for white, *p* = 0.02), smoking (higher in non‐smokers, *p* = 0.05) estimated fitness (positively correlated, *p* < 0.001) and PASAT arousing rating (positively correlated, *p* = 0.03). Correlation *r*‐values are shown in Table S1.

### 2.6 Primary Analyses: Predicting Reactivity From Exercise Dependence Adjusting for Covariates

After adjusting for the relevant covariates identified above, the linear associations between EABQ, EBQ and EDQ scores and cardiovascular reactivity remained non‐significant (see Table 2).

In the subsample, after adjusting for the relevant covariates, the association for EBQ remained significantly negatively associated with SBP reactivity (β = −0.27, *p* = 0.02, Δ*R* ^2^ = 0.06) and DBP reactivity (β = −0.22, *p* = 0.048, Δ*R* ^2^ = 0.04) such that higher exercise dependence scores related to lower BP reactivity. For the EDQ, the previous trend with SBP reactivity was now non‐significant (*p* = 0.16). All other previously non‐significant associations remained so.

### 2.7 Secondary Replication Analyses

To replicate Heaney et al. (2011), young female participants were divided into an extreme exercise‐dependent group and an extremely non‐dependent group, based on the 10 highest and lowest scorers on the EABQ; due to identical scores this resulted in 11 in the exercise dependent group and 10 in the non‐dependent group. Comparison between the present extreme group scores and Heaney et al. (2011)'s are shown in Table 3.

**TABLE 3:** Comparisons between groups extreme on exercise dependence based on EABQ highest and lowest scores.

	Present sample—exercise dependent (*n* = 11)	Present sample—non‐dependent (*n* = 10)	Heaney et al. (2011) Exercise dependent (*n* = 10)	Heaney et al. (2011) non‐ dependent (*n* = 10)
EABQ score	50.2 (8.04)	5.0 (3.33)	48.6 (7.93)	1.3 (1.77)
EDQ score	136.5 (20.50)	76.1 (15.77)	145.3 (12.31)	69.6 (11.52)
EBQ score	1299.3 (308.31)	634.8 (381.02)	1204.0 (418.87)	443.0 (226.77)
SBP reactivity (mmHg)	10.6 (6.03)	6.7 (8.52)	9.8 (6.86)	8.1 (6.52)
DBP reactivity (mmHg)	10.0 (5.20)	6.9 (9.86)	6.0 (6.12)	5.7 (5.67)
HR reactivity (bpm)	14.9 (9.19)	9.1 (7.09)	7.9 (4.61)	15.5 (9.58)

In these analyses, there were no significant group differences in reactivity outcome and the exercise‐dependent group showed slightly higher reactivity. Adjustment for relevant potential confounders did not change these results, although there was a trend for the exercise dependent group to have higher HR reactivity (*p* = 0.06) than those in the non‐dependent group on the EABQ.

### 2.8 Sensitivity Analysis

Next, as the EDQ has a cut‐off score of > = 116 which is recommended to indicate individuals who are exercise dependent, a new variable was created to reflect those above (*n* = 53 [33%], 36 females) and below this cut‐off. Comparing the stress reactivity of these groups showed that the DBP reactivity of the exercise‐dependent group was lower, *F*(1,158) = 3.97, *p* = 0.048, η^2^ = 0.024 and this effect was stronger after adjustment for confounders, *F*(1,151) = 6.45, *p* = 0.012, η^2^ = 0.041.

Using the EDQ cut‐off in the subsample (*n* = 36 classed as exercise dependent) and comparing the stress reactivity of these groups only showed a non‐significant trend for SBP reactivity (*p* = 0.08) such that the exercise dependent group showed lower reactivity, but this remained non‐significant following adjustment (*p* = 0.09).

## 3 Discussion

This extension and replication study provided partial support for our hypothesis that there would be a linear negative relationship between exercise dependence and physiological reactivity to stress. However, this pattern was evident only in the young female subsample (most comparable to the prior study) and was observed primarily for EBQ and EDQ scores, which capture dependence‐like beliefs and behaviors, in relation to SBP and DBP responses. Analyses attempting to replicate Heaney et al.'s study by comparing extreme groups on the EABQ, which captures exercise salience and attitudes, revealed no significant differences in reactivity; however, using the EDQ cut‐off to delineate exercise dependence showed some indications of blunted BP reactivity in exercise dependent individuals.

The present findings partially extend those of Heaney et al. (2011) by showing that exercise dependence relates to lower acute stress reactivity linearly in young women, not just in an extreme group. However, there are some important differences. First, the present negative associations emerged mainly for the EBQ measure, with some for the EDQ (including using the cut‐off for presence/absence of exercise dependence), but not for the EABQ. Second, the reactivity differences were observed for SBP and DBP reactivity, whereas in the previous study these emerged for HR and cortisol reactivity. Further, the attempt to replicate the previous findings by assigning extreme groups based on EABQ score revealed no significant differences in cardiovascular reactions to stress; indeed, if anything the exercise‐dependent group showed indications of slightly higher reactivity.

Potentially, the differences across the exercise dependence measures are due to the means and distributions of the different samples across the two studies. As Table 3 shows, this sample was somewhat less extreme than the previous; the present non‐dependent group is not as close to zero on all measures as Heaney et al.'s non‐dependent group, making extreme group scores harder to detect. Thus, in the young female subsample, the EBQ score distribution may have provided a clearer gradient of exercise dependence, increasing the likelihood of detecting associations for this measure. Further, in the replication analyses, when the present subsample was split by extremes on the EABQ, one individual did not additionally meet the criterion for exercise dependence on the EDQ, whereas in the previous study, they did and one individual in the non‐dependent group based on the EABQ had a high EDQ score of 101. This may indicate that although the groups differed markedly in self‐reported exercise attitudes or exercise salience (EABQ), differences in addictive exercise beliefs/behaviors captured by the EBQ and EDQ were less pronounced. Consistent with this interpretation, reactivity differences were more apparent when dependence was operationalized using the EDQ criterion rather than EABQ‐based extreme groups.

In addition, the cardiovascular outcomes which showed evidence of lower responses among those with higher exercise dependence scores differed from those previously. In the present study, the effects were most consistent for blood pressure, whereas Heaney et al. (2011) found the significant differences in HR and cortisol. Appreciably, cortisol reactivity in the present subsample was not significant or in either subgroup of exercise dependence, so direct comparisons cannot be made. The different findings for blood pressure versus HR between studies have previously been explained as different variables of interest potentially having different mechanisms of effect on reactivity, for example, cardiac versus more vascular (Carroll et al. 2008). However, with the present study repeating the same psychosocial measures as previously, this does not seem likely. Potentially, the differences here are more due to variations in the magnitude of the stress response in this larger sample compared to the previous sample. For example, in the present study the mean (SD) SBP, DBP and HR reactivity were higher than that observed by Heaney et al. (2011) (SBP: 8.7 [6.50], DBP: 5.3 [5.79] and HR reactivity: 11.7 [8.29]) but the differences were far more marked for SBP and DBP, perhaps representing a shift in response channel from cardiac to more vascular in the present sample. Alternative explanations might be differences in the fitness level of participants between studies or measurement sensitivity. On this latter point, Heaney et al.'s study, average values per phase were calculated from physiological measurements taken three times during baseline and four times during stress, whereas in the present study, as we also had continuous measurement by finger noninvasive blood pressure (NIBP) and pulse, we only took discontinuous measures at 2 and 6 min of each phase. Unfortunately, due to equipment failure resulting in excessive missing data, our plan to focus analyses on the NIBP data was not possible, so here we only report on the full sample with reactivity data from the sphygmomanometer.

In other measurable ways, the reactivity of the present sample did not differ too much from previous similar research. For example, habitual smokers were found to exhibit lower SBP reactivity under acute stress here compared to non‐smokers as has been shown previously (Al'Absi 2018; Al'Absi et al. 2003; Ginty et al. 2014, 2016; Girdler et al. 1997; Kirschbaum et al. 1993, 1994; Rohleder and Kirschbaum 2006; Roy et al. 1994; Sheffield et al. 1997; Straneva et al. 2000) and alcohol drinkers were also found to exhibit lower HR reactivity here than non‐drinkers, consistent with previous reports of lower HR or cortisol reactivity in those with alcohol dependence (Jones et al. 2013; Lovallo 2006; Panknin et al. 2002; Sorocco et al. 2006). Overall, the relationships between substance addiction, like smoking and low reactivity have been confirmed by two large cohort studies; the West of Scotland 20‐07 study (Phillips, Der, Hunt, and Carroll 2009) and Dutch Famine Birth Cohort Study (Ginty et al. 2014), as well as several smaller laboratory studies. Potentially, the effects for substance addictions are more consistent and robust than for non‐substance addictions measured through self‐report rather than presence/absence of substance consumption. The relationship between other self‐reported non‐substance addictions or dependencies, including more recent ones such as screen or device addictions, could be further studied to test this.

### 3.1 Limitations

First, the first set of analyses in this study included a mixed sex sample, which might have influenced the initial null results as exercise dependence is more likely to occur in women (Bamber et al. 2000, 2003), thus, a female sample might be expected to have higher scores. However, it was possible to recruit enough young females within this sample to also conduct analyses to extend and replicate previous work, which was one of the aims. Second, although the full sample was more diverse, there were not equal numbers of males and females and the age range was still relatively restricted as all participants were university students, thus homogeneity was high. However, the proportion of Sport undergraduate and postgraduate students was high, so there was similarity to the samples of Heaney et al.'s (2011) research which meant this was a close replication and extension. Third, as mentioned above, we had hoped to include continuous BP and HR data across each phase and use this to calculate reactivity, but equipment failure prevented this. Gladly, we still had repeated cardiovascular measures during each phase with which to compute reactivity values and conduct our planned analyses. Fourth, cortisol reactivity was not significant overall in this sample, which could reflect the smaller subsample with measures or different profile of response in the present sample compared to the previous study, or testing times. The previous study tested only at 15:00 or 16:30 h whereas, to maximize sample size, the present study tested from 13:00 h onwards, so although this still avoided the morning due to diurnal variation that is the steeper decline in the morning compared to the afternoon, it was not as strict and a student sample with potentially later waking times might have meant the potential stress response was somewhat masked by diurnal variation in cortisol levels. Further, this study used subjective methods to estimate fitness and self‐reported exercise levels. Due to time restrictions, the objective assessment of cardiorespiratory fitness levels via sub‐maximal testing or physical activity levels via accelerometer measurements could not be conducted. Research has indicated that objective measurement and subjective measurement are not interchangeable (Emaus et al. 2010), but inclusion as a subjective variable to control for is preferable to ignoring potential confounding by these important variables. One final important consideration is that exercise dependence differs from most other behavioral or substance‐related addictions because the behavior itself produces positive physiological adaptations resulting in better physical fitness and cardiovascular function. This makes it difficult to discern whether the altered cardiovascular stress reactivity observed here is a result of addiction/dependence processes in the brain or simply an adaptation associated with high levels of exercise engagement. It could be argued that we accounted for this through adjustment for estimated fitness, but adjusting for objective cardiorespiratory fitness would be more rigorous. This potential confounding by physical fitness makes direct comparison between exercise dependence and other addictions or dependencies difficult. However, a better understanding of the mechanisms underlying low reactivity in those with exercise dependence could be investigated through brain imaging to see whether individuals with exercise dependence also show dysregulation in response to stress in the brain areas associated with motivated behaviors as evident in other samples with low reactivity or its correlates, for example, depression.

### 3.2 Future Directions

Future research directions in this area could importantly include non‐student older female samples who may potentially be at higher risk of pathological exercise dependence and its effects due to possible longer‐term engagement in exercise‐dependent behaviors. Further, it would be of interest to explore whether other group differences in reactivity previously shown between extreme groups are only observed between extreme scorers rather than linear associations across the range, for example, with disordered eating and childhood adversity. A better understanding of the nature of these mechanisms would help to determine whether a lowered ability to respond to stress is likely to be adaptive or a marker of higher‐level dysregulation—something which could also be explored through brain imaging, as mentioned above. If the extent of alteration in capacity to respond to stress is deemed to be dysregulation at the brain level, this could form the basis for interventions attempting to reverse these changes, using cardiovascular stress reactivity as a noninvasive peripheral marker.

## 4 Conclusion

This study demonstrated that the degree of exercise dependence across a range of scores shows significant linear associations with cardiovascular reactivity to acute psychological stress only in younger females (not the full mixed‐sex sample). This pattern suggests that any physiological correlates of exercise dependence may be more detectable in young women, consistent with prior work and the higher prevalence of exercise dependence in females. Comparing exercise dependence extreme groups in an attempt to replicate previous analyses (Heaney et al. 2011) did not show significant differences in reactivity but this might be explainable by the EABQ measure used to create the groups and the scores in the present sample on this, because a sensitivity analysis using the EDQ cut‐off for dependence showed some evidence of lower DBP reactivity. These effects generally withstood adjustment for stress task performance and cardiovascular fitness. Future research could explore whether reactivity differences are only evident between groups extreme in other addictions/dependencies or across the range of dependence (e.g., for cigarette/vape use or alcohol intake) as well as for non‐substance dependencies such as disordered eating.

## Author Contributions

**Yuan Tian:** data curation, investigation, formal analysis, visualization, writing – original draft, writing – review and editing. **Siobhán Howard:** writing – review and editing, supervision. **Jenni Connelly:** investigation, supervision, writing – review and editing. **Anna C. Whittaker:** conceptualization, methodology, data curation, investigation, validation, formal analysis, supervision, visualization, project administration, resources, writing – original draft, writing – review and editing. **Aiden J. Chauntry:** writing – review and editing. **Jennifer L. J. Heaney:** methodology, writing – review and editing.

## Conflicts of Interest

The authors declare no conflicts of interest.

## Supporting information


**Table S1:** Covariate correlations (r) or group differences (F) for covariates explored for both the full sample and sub‐sample analyses. Bolded values are significant at *p* < 0.05.

## Data Availability

The data that support the findings of this study are available from the corresponding author upon reasonable request. Upon manuscript acceptance the data and code will be archived alongside it in the University of Stirling data STORRE.
