# Supplementary material for: Exercise Dependence and Cardiovascular Reactivity to Acute Psychological Stress: An Extension and Replication Study
Source: Psychophysiology. 2026 Aug 25;63(8):e70389. doi: 10.1111/psyp.70389 (PMC13507582; doi:10.1111/psyp.70389)
Supplement: Supplementary file 1 [file PSYP-63-e70389-s001.docx]

**Supplementary Table 1: Covariate correlations (r) or group differences (F) for covariates explored for both the Full sample and Sub-sample analyses. Bolded values are significant at p<.05.**

|  | r / F | | | | | |
| --- | --- | --- | --- | --- | --- | --- |
|  | SBP reactivity | DBP reactivity | HR reactivity | EABQ | EBQ | EDQ |
| **Full Sample** |  |  |  |  |  |  |
| Baseline SBP | -.14 | -.09 | .003 |  |  |  |
| Baseline DBP | -.10 | **-.35** | -.02 |  |  |  |
| Baseline HR | -.11 | **-.17** | **-.25** |  |  |  |
| PASAT score | **.19** | -.01 | .08 | **.24** | .14 | .10 |
| Age | **.21** | .15 | .01 | -.08 | -.12 | **-.22** |
| BMI | .11 | -.04 | -.06 | .02 | .08 | -.08 |
| SCOFF total | -.10 | -.02 | -.04 | **.17** | **.21** | **.17** |
| Estimated Fitness | -.13 | -.19 | **-.16** | **.39** | **.18** | **.34** |
| PASAT Difficulty | -.07 | -.05 | -.01 | -.03 | .02 | -.05 |
| PASAT Stressfulness | -.13 | .01 | **.17** | .01 | .11 | **.17** |
| PASAT Arousing | .07 | **.17** | .11 | **.21** | **.30** | **.24** |
| PASAT Performance | **.21** | .15 | -.04 | .01 | -.04 | -.09 |
| PASAT Confusing | -.04 | .03 | -.10 | **-.24** | **-.28** | **-.20** |
| PASAT Engaging | .08 | -.03 | .06 | .09 | .02 | -.01 |
| PASAT Embarrassing | -.01 | .03 | -.01 | -.11 | .02 | .04 |
| Gender | 1.85 | 0.13 | **5.37** | **9.18** | 1.50 | 0.01 |
| Ethnicity | 0.01 | 0.56 | 0.40 | 1.05 | 3.10 | **4.53** |
| Smoking status | **4.61** | 0.70 | 0.04 | **4.16** | 0.72 | **5.88** |
| Drinks alcohol | 1.71 | 0.28 | **4.93** | 0.50 | 1.43 | 0.09 |
|  |  |  |  |  |  |  |
| **Sub-Sample** |  |  |  |  |  |  |
| Baseline SBP | -.06 | -.13 | .10 |  |  |  |
| Baseline DBP | -.04 | **-.31** | .02 |  |  |  |
| Baseline HR | -.08 | -.15 | **-.25** |  |  |  |
| PASAT score | **.23** | .02 | .18 | .16 | .12 | .11 |
| Age | .18 | .15 | .06 | -.18 | -.19 | **-.21** |
| BMI | .06 | -.05 | -.06 | .03 | .14 | -.08 |
| SCOFF total | -.13 | -.03 | -.09 | .14 | **.24** | .13 |
| Estimated Fitness | -.16 | -.12 | -.04 | **.36** | .16 | **.46** |
| PASAT Difficulty | -.12 | -.02 | -.04 | -.04 | .01 | -.08 |
| PASAT Stressfulness | -.18 | -.08 | .13 | .16 | .17 | .14 |
| PASAT Arousing | -.06 | -.02 | .08 | .14 | **.28** | **.22** |
| PASAT Performance | .12 | .09 | -.05 | -.11 | -.08 | -.14 |
| PASAT Confusing | -.03 | .10 | -.09 | -.15 | **-.27** | **-.12** |
| PASAT Engaging | .08 | .04 | .09 | -.02 | -.06 | -.07 |
| PASAT Embarrassing | .10 | .04 | .04 | .001 | .08 | .08 |
| Ethnicity | 0.20 | 1.84 | 1.76 | 2.56 | **6.49** | **5.91** |
| Smoking status | **3.10** | 0.13 | 042 | 1.27 | 0.53 | **3.98** |
| Drinks alcohol | 3.56 | 3.21 | **5.76** | 0.85 | 0.79 | 0.58 |
